# Single Molecule Investigation of Ag^+^ Interactions with Single Cytosine-, Methylcytosine- and Hydroxymethylcytosine-Cytosine Mismatches in a Nanopore

**DOI:** 10.1038/srep05883

**Published:** 2014-08-08

**Authors:** Yong Wang, Bin-Quan Luan, Zhiyu Yang, Xinyue Zhang, Brandon Ritzo, Kent Gates, Li-Qun Gu

**Affiliations:** 1 Department of Biological Engineering and Dalton Cardiovascular Research Center University of Missouri, Columbia, MO 65211, USA; 2 Computational Biology Center, IBM T. J. Watson Research, Yorktown Heights, NY 10598, USA; 3 Department of Chemistry, University of Missouri, Columbia, MO 65211, USA; 4 These authors contributed equally to this work.

## Abstract

Both cytosine-Ag-cytosine interactions and cytosine modifications in a DNA duplex have attracted great interest for research. Cytosine (C) modifications such as methylcytosine (mC) and hydroxymethylcytosine (hmC) are associated with tumorigenesis. However, a method for directly discriminating C, mC and hmC bases without labeling, modification and amplification is still missing. Additionally, the nature of coordination of Ag^+^ with cytosine-cytosine (C-C) mismatches is not clearly understood. Utilizing the alpha-hemolysin nanopore, we show that in the presence of Ag^+^, duplex stability is most increased for the cytosine-cytosine (C-C) pair, followed by the cytosine-methylcytosine (C-mC) pair, and the cytosine-hydroxymethylcytosine (C-hmC) pair, which has no observable Ag^+^ induced stabilization. Molecular dynamics simulations reveal that the hydrogen-bond-mediated paring of a C-C mismatch results in a binding site for Ag^+^. Cytosine modifications (such as mC and hmC) disrupted the hydrogen bond, resulting in disruption of the Ag^+^ binding site. Our experimental method provides a novel platform to study the metal ion-DNA interactions and could also serve as a direct detection method for nucleobase modifications.

In DNA duplexes, silver ions specifically interact with C-C mismatches^1^,^2^,^3^,^4^, while mercury ions specifically interact with T-T mismatches^5^,^6^,^7^,^8^. These interactions that strongly stabilize DNA duplexes have been extensively studied recently^9^, but the nature of coordination of Ag^+^ with C-C mismatches is not clearly understood^4^,^10^,^11^,^12^. Considering that cytosine (C) modifications such as 5-methylcytosine (mC) and 5-hydroxymethylcytosine (hmC) are important epigenetic markers associated with gene expression and tumorigenesis^13^,^14^,^15^, we were motivated to explore the interactions of Ag^+^ with a DNA duplex containing a single C-C, C-mC or C-hmC mismatch in the alpha-hemolysin nanopore (α-HL). The α-HL has a nanocavity (2.6 nm opening with a 1.4 nm constriction site) that can capture and hold the DNA duplex (Supplementary Figure S1), providing an ideal platform for studying both the C-Ag-C interaction and how cytosine modifications change this interaction. In a nanopore experiment, an electric field drives charged molecules through a nanometer-scale pore that spans an insulating membrane, which separates two aqueous solutions. The baseline ionic current through the pore is transiently blocked by larger macromolecules (such as DNA) that enter the pore. The ion current through a nanopore is sensitive to target molecules that interact with the pore, therefore different molecular states can be electrically clarified from characteristic changes in the nanopore current. The α-hemolysin nanopore has been studied for DNA sequencing^16^,^17^,^18^, various single-molecule detections^19^,^20^,^21^ and biomolecular interactions^22^,^23^,^24^,^25^.

In previous nanopore studies^26^,^27^,^28^,^29^, researchers have found that C, mC or hmC can be recognized by immobilizing the DNA with streptavidin^28^, or by chemical modifications^26^ in α-HL. When in a solid-state nanopore, it was found that DNA duplexes containing mC and hmC can be discriminated^29^, and by using methylated CpG binding proteins, C and mC themselves could also be discriminated^27^. Several other methods can be used to distinguish hmC, mC, and C bases with chemical modifications via sequencing^30^,^31^,^32^. In this report, we described a unique nanopore sensor that can *directly* discriminate cytosine and cytosine modifications *simultaneously* (evidenced by ionic current signals such as dwell times (t_off_, Supplementary Figure S1) and residual currents (Supplementary Figure S1) without modifications. The key principle of this novel method for cytosine modifications determination is the fact that Ag^+^ stabilizes a C-C containing DNA duplex, which was confirmed in the nanopore for the first time. By molecular dynamics (MD) simulations, we found that cytosine modifications such as mC and hmC disrupted both the hydrogen bonds and Ag^+^ interactions, which subsequently affected DNA-Ag^+^ stability (in the term of rate of dissociation).

## Results

**Figure 1:**
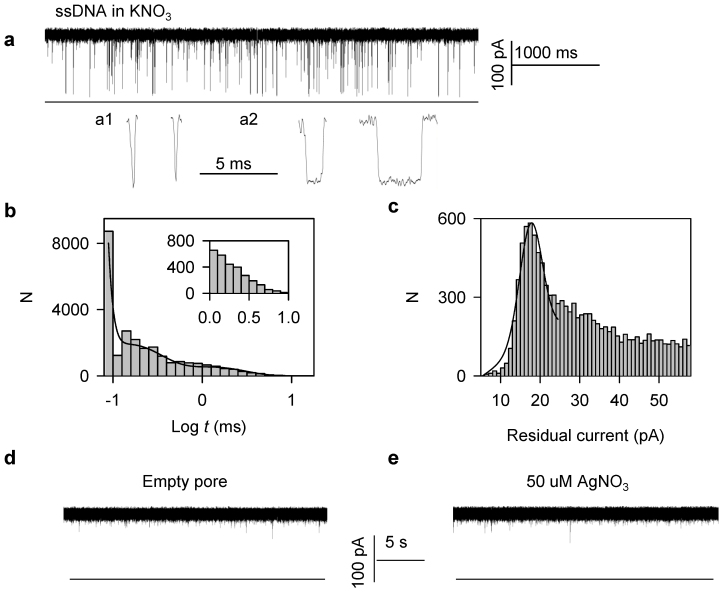
The ssDNA P interacts with the nanopore. (a) The representative current trace recorded at 150 mV. Two types of events were identified: a1: spike-like current profile which last about 200 us and a2: rectangular-like current profile which last about 1 to 10 ms. (b) The histogram of the dwell time in Log form. The long events (>10^0^ = 1 ms) were easily identified. (c) The histogram of residual currents when the ssDNA P interacts with the nanopore. The nanoporecurrent traces of the empty pore (d) and with the addition of 50 uM AgNO_3_ (e) recorded at 150 mV in 1 M KNO_3_.

The study involved three 16-nt AT rich ssDNAs as the targets, which contain a cytosine (T_C_), 5′-methylcytosine (T_mC_) and 5′-hydromethylcytosine (T_hmC_) at the 10^th^ nucleotide (5′ → 3′), respectively (Table 1). Their common probe, P, contains a cytosine at the corresponding position, such that when P is hybridized with the three targets, their hybrids P**·**T_C_, P**·**T_mC_ and P**·**T_hmC_, form a C-C, C-mC and C-hmC mismatched base-pair respectively. Since Ag^+^ was tested in the experiments, we could not use KCl buffer due to AgCl precipitation. Therefore, we first tested how the single-stranded DNA (ssDNA) P (Figure 1) interacts with the nanopore in KNO_3_ solution. Short (<1 ms) and long events in the range of 1–10 ms were easily identified (Figure 1a,b). The residual current also has a wide distribution, with a peak at 17.4 ± 0.84 pA (Figure 1c). Others have previously noted that KNO_3_ has unknown effects on DNA translocation and some extraordinary long events were seen, with about 10-fold lower occurrence rate constant (*K*_on_) of ssDNA in KNO_3_ than in the KCl buffer^8^, as well as in certain cations such as Li^+^ 33 and ion liquid^34^. In order to ensure the ssDNA interactions were excluded, we only considered events longer than 10 ms as the DNA duplexes interact with the nanopore. A control experiment demonstrated that Ag^+^ itself does not affect the open pore current (Figure 1d.e). The positively charged Ag^+^ is driven away from the nanopore by the applied voltage.

### Ag^+^ stabilizes a DNA duplex with C-C mismatches

**Figure 2:**
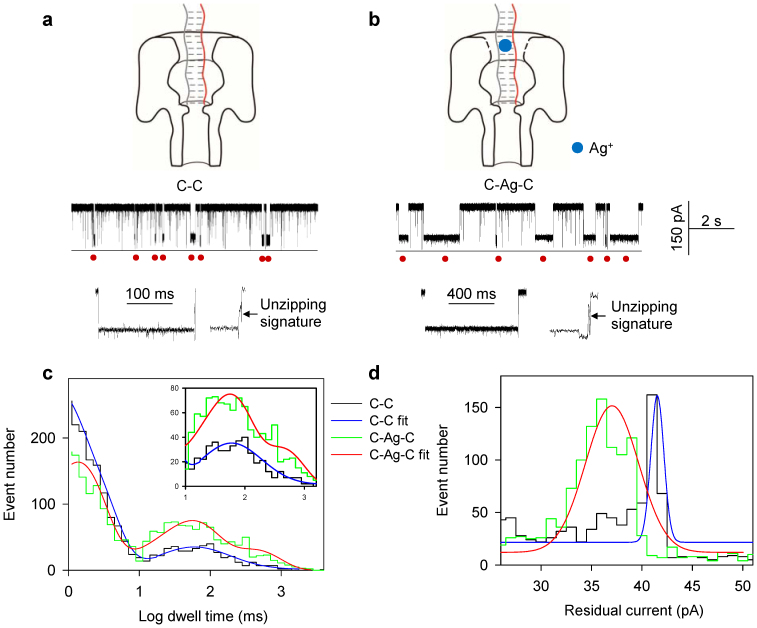
Ag^+^ stabilizes DNA duplex containing C-C mismatches. (a) The capturing of C-C duplex (ssDNA T_C_ hybridized with P) in the nanopore. (b) The capturing of C-C duplex with the addition of Ag^+^. (c) The histogram of the dwell time in Log form. The C-C generated a single peak of 59 ± 5 ms (blue). The C-Ag-C generated two peaks of 51 ± 6 ms and 384 ± 12 ms (red), which increased the dwell time by 6.5 fold compared to the C-C duplex. (d) The histogram of residual currents. The C-C generated a single peak of 41.5 ± 0.4 pA (blue); The C-Ag-C generated a peak of 36.8 ± 0.2 pA. The difference was 4.7 ± 0.45 pA between C-C and C-Ag-C. The red circles indicate the capturing of DNA duplexes. The enlarged single traces in a and b demonstrated the DNA duplex dissociation signature with an ending spike. Recordings were made at 150 mV.

The addition of Ag^+^ increases the stability of dsDNA containing a C-C mismatch, which leads to an increase in the complex's dwell time within the nanopore (Figure 2a,b). We can see that ssDNAs (dwell time <10 ms) and dsDNAs (dwell time >10 ms) were well separated (Figure 2c). For details on the probe screening process, please refer to the supplementary information (Supplementary Note S1). The events with an ending spike^35^,^36^,^37^,^38^ were identified (Figure 2a,b enlarged single current traces), indicating the DNA duplex capturing and dissociation (See Supplementary Note S2 for detailed description). The difference in dwell time provides a key differentiator between C-C and C-Ag-C. In detail, P**·**T_C_ hybrid (C-C) yielded the dwell time distribution with a peak at 59 ± 5 ms (Figure 2c, blue), while C-Ag-C yielded a dwell time distribution with the first peak at 51 ± 6 ms and the second peak at 384 ± 12 ms (Figure 2c, red). Molecular dynamics (MD) simulations indicate that hydrogen bonds are alternatively formed between N4_A_-N3_B_ and N3_A_-N4_B_ atoms (simulations described in details below), and there is a 2.6-fold difference in binding energy bewteen these two conformations. This difference in binding energy could be the reason that we observed two dwell time distributions peaks. This second peak demonstrates dwell times with C-Ag-C that are 6.5-fold longer than those with C-C (Figure 2c). We interpret that the prolonged blocking events are due to the binding of Ag^+^ to the C-C mismatch in the P**·**T_C_ hybrid. As reported previously, the binding of Ag^+^ forms a C-Ag-C bridge base pair that stabilizes the P**·**T_C_ complex^1^,^2^,^3^,^4^, resulting in an extended dwell time under the same holding potential. The Ag^+^ effect is equivalent to an increase in dsDNA hybridization energy, which was calculated to be 3.8 ± 0.5 kJ**·**mol^−1^ using Δ*E* = *RT*ln(*τ*_+Ag_/*τ*_−Ag_), where *τ*_−Ag_ and *τ*_+Ag_ are block durations before and after the addition of Ag^+^. We also found a decrease in residual current after the addition of Ag^+^. They are 41.5 ± 0.4 pA (without Ag^+^) and 36.8 ± 0.2 pA (with Ag^+^), respectively (Figure 2d). The change is 4.7 ± 0.45 pA (by error propaganda equation). The hydrated radius of Ag^+^ is 0.34 nm^39^, and as a result, the substantial radius of Ag^+^ in complex with the DNA blocks more current flow. Thus it is reasonable to see a deeper current blockage for DNA with Ag^+^.

We further compared the equilibrium dissociation constant (*K_d_*) for P**·**T_C_ in the absence and in the presence of Ag^+^. We have derived an expression to obtain *K_d_* from the block frequency (See Supplementary S1: nanopore measurement of double-stranded DNA equilibrium constant). The expression is *K_d_* = (*f_ss_*/*k_on_*)^2^/2([ssDNA]_0_-*f_ss_*/*k_on_*), where *k_on_* is the average ssDNA (P or T_C_) capture rate in the nanopore, and *f_ss_* is the total frequency of blocks generated by unhybridized ssDNA (P and T_C_) in the mixture. We found that Ag^+^ can decreases *f_ss_* from 6.52 ± 0.38 s^−1^ to 4.10 ± 0.19 s^−1^. This decrease of *f_ss_* is also confirmed by an increase of the t_on_ (See Supplementary Figure S1 for definition) for 1.6-fold (Supplementary Figure S2). We found a decrease of *K_d_* from 0.12 ± 0.01 μM^−1^ to 0.04 ± 0.004 μM^−1^ (Table 2), suggesting that the stabilization of P**·**T_C_ by Ag^+^ shifts the equilibrium of the reaction P + T_C_↔P**·**T_C_ toward the product P**·**T_C_. The decrease of *K_d_* is expected to increase the melting temperature (*T_m_*). Indeed, the UV measurement shows that *T_m_* for the mixture of P/T_C_ in 1 M KNO_3_ increased from 28.5 ± 0.6°C (without Ag^+^) to 43.5 ± 0.6°C (with addition of Ag^+^), confirming the equilibrium shift toward the duplex formation due to the Ag^+^ stabilization of dsDNA. Overall, the C-Ag-C bridge-pair functions as an interstrand lock, or SilverLock, that greatly stabilizes dsDNA hybridization. The resulting nanopore signature for SilverLock can identify a single C-C mismatch in a dsDNA.

### Weak interaction of Ag^+^ with a DNA duplex containing mC-C mismatches

**Figure 3:**
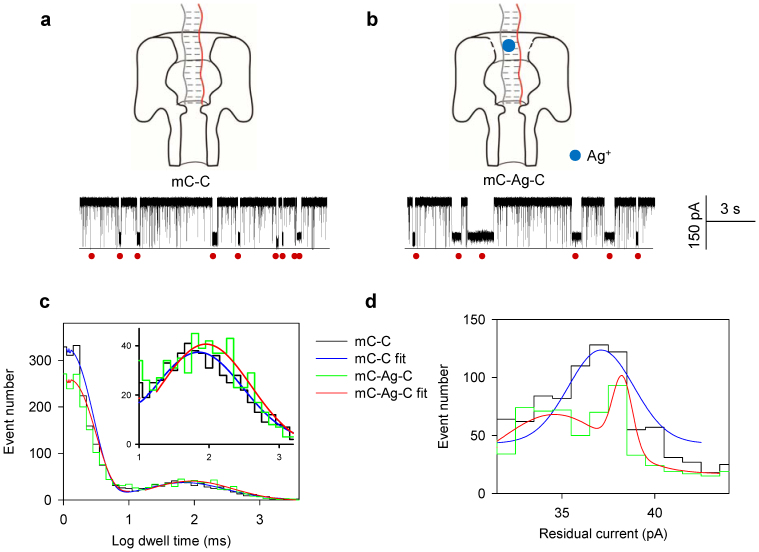
Weak interaction of Ag^+^ with a DNA duplex containing mC-C mismatches. The representative current traces of mC-C (a) and mC-Ag-C (b) capturing. (c) The histogram of the dwell time in Log form. The mC-C generated a single peak of 69 ± 6 ms (blue). The mC-Ag-C generated a single peak of 92 ± 10 ms (red), which increased the dwell time by 1.3 fold. (d) The histogram of residual currents. The mC-C generated a single peak of 37.4 ± 0.7 pA (blue). The mC-Ag-C generated two peaks of 33.9 ± 0.8 pA and 38.1 ± 0.8 pA (red). The difference was 3.5 ± 1.1 pA between mC-C and the first peak of mC-Ag-C. The red circles indicate the capturing of DNA duplexes. Recordings were made at 150 mV.

The addition of Ag^+^ also increases the stability of dsDNA containing an mC-C mismatch (probe P is hybridized with the target T_mC_, their hybrid P**·**T_mC_ forms a single C-mC mismatch), though the increase in dwell time is less than those for C-C (Figure 3a,b). We found that P**·**T_mC_ yielded a dwell time distribution peaked at 69 ± 6 ms (Figure 3c, blue), while P**·**T_mC_ with Ag^+^ yielded a peak at 92 ± 10 ms (Figure 3c, red), which represents a 1.3-fold increase in dwell time, corresponding to a 0.53 ± 0.07 kJ**·**mol^−1^ increase of the energy for dsDNA dehybridization. This energy increase is lower than the 3.8 kJ**·**mol^−1^ for dsDNA containing a C-C mismatched base pair bound with Ag^+^, suggesting that the effect of Ag^+^ on stabilization of dsDNA with a C-mC mismatch is much weaker than that with a C-C mismatch.

For residual currents, P**·**T_mC_ yielded a peak at 37.4 ± 0.7 pA and P**·**T_mC_ with Ag^+^ yielded two residual current peaks at 33.9 ± 0.8 pA and 38.1 ± 0.8 pA (Figure 2d). The difference was about 3.5 ± 1.1 pA between the peak of mC-C and the first peak of mC-Ag-C (Figure 2d). This suggests that the interaction between mC-C and Ag^+^ was weaker than that between C-C and Ag^+^ (See Supplementary Note S3 for discussion).

### No observable interaction of Ag^+^ with a DNA duplex containing hmC-C mismatches

**Figure 4:**
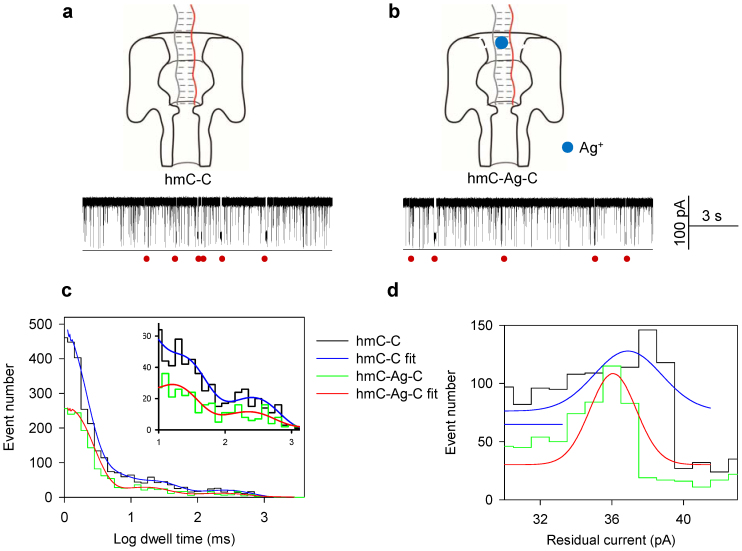
No observable interaction of Ag^+^ with a DNA duplex containing hmC-C mismatches. The representative current traces of hmC-C (a) and hmC-Ag-C (b) capturing. (c) The histogram of the dwell time in Log form. The hmC-C generated a single peak of 19.6 ± 1 ms (blue). The hmC-Ag-C generated a single peak of 17.3 ± 1 ms (red). (d) The histogram of residual currents. The hmC-C generated a single peak of 36.3 ± 0.95 pA (blue); The hmC-Ag-C generated a single peak of 36.2 ± 0.71 pA (red). The difference was 0.1 ± 1.19 pA. The red circles indicate the capturing of DNA duplexes. Recordings were made at 150 mV.

We also measured the effect of Ag^+^ on the dsDNA containing a C-hmC mismatched base pair (probe P is hybridized with the target T_hmC_, their hybrid P**·**T_hmC_ forms a single C-hmC mismatch). The addition of Ag^+^ does not appear to affect the stability of dsDNA containing an hmC-C mismatch, though dwell time is lower than those for C-C and mC-C mismatches (Figure 4). We found that P**·**T_hmC_ yielded a dwell time distribution which is very similar to that of P**·**T_hmC_ with Ag^+^ (Figure 4a,b,c). The hmC-C yielded a dwell time distribution peaked at 19.6 ± 1 ms (Figure 4c, blue), while hmC-Ag-C yielded a peak at 17.3 ± 1 ms (Figure 4c, red). For residual current, P**·**T_hmC_ yielded a peak at 36.3 ± 0.95 pA and P**·**T_hmC_ with Ag^+^ yielded a similar peak at 36.2 ± 0.71 pA (Figure 4d). The difference was 0.1 ± 1.19 pA. Overall, these data demonstrate that hmC-C mismatches are less stable than mC-C or C-C mismatches. Therefore, the presence of Ag^+^ seems to have little effect on the C-hmC mismatch.

Besides the dwell time, the addition of Ag^+^ decreased the residual current at different degrees for the tested DNA duplexes, which provides the second key differentiator to discriminate C,mC and hmC (Supplementary Figure S3). We also found that Ag^+^ does not interact with ssDNAs T_C_, T_mC_ or T_hmC_ (Supplementary Figure S4).

### Molecular dynamics (MD) simulations

**Figure 5:**
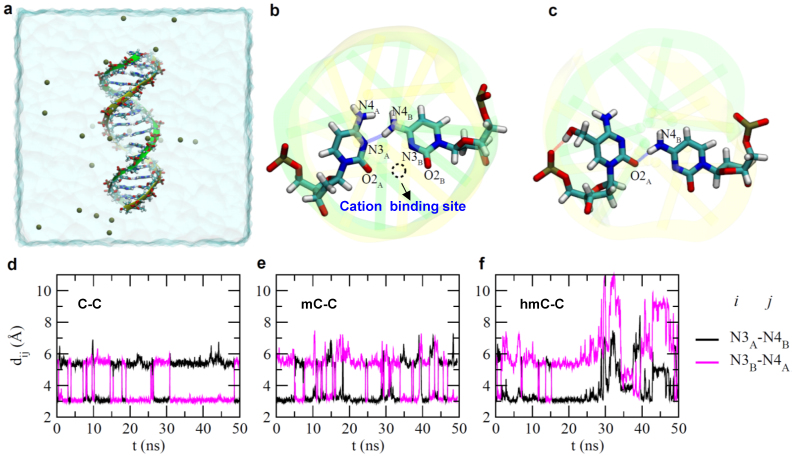
Molecular dynamics simulations of DNA duplex containing C-C, mC-C and hmC-C mismatches. (a) (LBQ is the creator of figure 5a). Side-view of the simulation system. The DNA duplex is in the “stick” presentation and two backbones are illustrated as yellow and green belts respectively. Potassium ions that neutralize the entire simulation system are shown as tan balls. Water in a cubic box (78.5 × 78.5 × 78.5 Å^3^) is shown transparently. (b) A snap-shot of pairing between two cytosine bases. The dashed circle highlights the binding site for a cation. (c) A snap-shot of hmC-C pairing before the pairing was broken. (d–f) Time-dependent distances between the N3 atom of one base and the N4 atom of the other base, in C-C(d), mC-C(e) and hmC-C(f) mismatches.

Molecular dynamics (MD) simulations of DNA duplexes containing these mismatches reveal how Ag^+^ may bind to the mismatches, as well as different coordination configurations between the mismatched bases (Supplementary Note S4 for simulation description). As shown in Figure 5a, a DNA duplex, with the same sequence as that in experiment was solvated in an electrolyte. The C-C base pairing was formed by the hydrogen bond between the N3 atom of one cytosine base (in the strand A) and the N4 atom of the other cytosine base (in the strand B) (Figure 5b). Besides the conformation shown in Figure 5b, another possible paring was formed by the hydrogen bond between N4_A_ and N3_B_ atoms (Supplementary Movie S1). The distances between N3 and N4 atoms of different bases, as shown in Figure 5d, indicate that hydrogen bonds are alternatively formed between N4_A_ and N3_B_ atoms and between N3_A_ and N4_B_ atoms. This type of pairing results in the formation of a binding site for a cation (Figure 5b). During the simulation, K^+^ ions were found in the binding site and the mean residence time for K^+^ was about 10 ns (Supplementary Movie S2). As confirmed in an independent MD simulation (Supplementary Figure s5, Movie S3), Ag^+^ can also enter the binding site and further stabilize the paring between mismatched C-C bases. Correspondingly, these simulations also indicate that the dwell time of the duplex with a Ag^+^ is longer (Figure 2c) due to the enhanced stability.

The simulations also reflect our experimental results for the differences in stability between the complexes. Figure 5e shows that, because of the switching between the two states of N4_A_-N3_B_ and N3_A_-N4_B_ (Figure 5b), the hydrogen bonds were formed and broken more frequently in mC-C compared to the C-C mismatch (Supplementary Movie S4). Additionally, the probability for having longer bond lengths was higher for the mC-C than for the C-C mismatch (Supplementary Figure S6). Therefore, these results suggest that the cation binding site in the mC-C duplex was less stable than in the C-C duplex, consistent with the experimental results that the dwell time of C-Ag-C was longer than mC-Ag-C duplex (Figure 2c, Figure 3c). Interestingly, for the duplex with the hmC-C, the base pairing was broken at about 25 ns during the simulation (Figure 5f, Supplementary Movie S5). Right before the breakage, Figure 5c shows that, because of the hydrogen bond between the hydroxyl group in the hmC base and the phosphate group, the hmC base rotated towards the backbone of the duplex. Such interaction could also be mediated by a water molecule (Supplementary Figure S7). Meanwhile, base pairing was formed between the O2 atom in the hmC base and the N4 atom of the C base. After the breakage, the hmC and C bases can temporarily form inter-strand base-stacking, which causes the breakage of a neighboring base-pair. Because the binding site falls apart in the duplex with the hmC-C mismatch, the effect of Ag^+^ on the dwell time should be negligible, as also demonstrated in nanopore experiments with hmC-C (Figure 4). Overall, this shows tight agreement between the theoretical and experimental results.

## Discussion

Studies have shown that Ag^+^ forms dinuclear complexes with cytosine and the complexes have been observed by X-ray diffraction. This study suggests that each of the methylcytosine residues doubly cross-linked by two Ag^+^ at the base binding sites N3 and O2^11^. Thermodynamic properties of C-Ag-C complexes were studied by isothermal titration calorimetry (ITC) and circular dichroism (CD) and the results suggest that the specific binding between the Ag^+^ and the single C-C mismatch was mainly driven by the positive dehydration entropy change of Ag^+^ and the negative binding enthalpy change from the bond formation between the Ag^+^ and the N3 positions of the two cytosine bases^4^,^10^. However, our MD simulation of C-Ag-C shows that Ag^+^ is dynamically coordinated between N3_A_ and O2_B_, or N3_B_ and O2_A_ (Figure 5b, Supplementary Figure S5). This finding suggests that the coordination of Ag^+^ in C-Ag-C complexes may have a different mechanism.

Different binding affinities for Ag^+^ ions with DNA duplexes containing C-C, mC-C or hmC-C could be explained in several ways. Firstly, the *T*m measurement demonstrates that Ag^+^ coordination raises the melting temperature through the stabilization effect of Ag^+^ on the C-C containing duplexes. Secondly, previous MD simulations found that H_2_O molecules have the highest affinity for hmC when compared to C and mC, which increases the rotation probability^29^. Our MD simulation revealed that the water molecule can mediate or directly interact with the phosphate group and the hydroxyl group in hmC. These results suggest a mechanism behind the lower stability of the base-pairing in hmC-C mismatches. Thirdly, using atomic force microscopy (AFM), studies have found that the persistence length follows the trend mC > C > hmC^29^, suggesting that hmC-containing DNA has the largest flexibility and least structural stability. Finally, the –OH group in hmC can chelate with the phosphate group^40^ which may prevent a stable hmC-Ag-C complex formation.

## Conclusion

Overall, we have demonstrated that chemical interactions between Ag^+^ and cytosine and its modifications could be applied to study C, mC and hmC differences. Without Ag^+^, the residual current follows C-C > mC-C > hmC-C (Figure 2,3,4d, blue; Figure S3a) and the dwell time follows mC-C > C-C > hmC-C (Figure 2,3,4c, blue). The residual current differences with the addition of Ag^+^ are C-C > mC-C > hmC-C (Figure 2,3,4d and Figure S3a,b). The dwell time differences (ratios) with the addition of Ag^+^ are also C-C > mC-C > hmC-C (Figure 2,3,4c). With these two key differentiators, we can discriminate C, mC and hmC bases. It is therefore concluded that the C-Ag-C mismatch is the most stable and the hmC-Ag-C is the least stable. This direct discrimination was successfully demonstrated without modification and amplification of target DNA. We also demonstrated that it is a dynamic coordination between Ag^+^ and C-C mismatches, which indicates a new binding mechanism. By utilizing the chemical interactions with metal ions, this approach might be extended to study other cytosine modifications, such as 5-formylcytosine (5fC) and 5-carboxylcytosine, and to investigate metallo-pair interactions^41^,^42^, including copper ion-stabilizing pyridine-2,6-dicarboxylate-pyridine mismatches and silver/mercury interacting with modified uracil pairs. Finally, it is also possible that a target fragment of a genomic sample could be obtained by a suite of restriction endonucleases. The target fragments can then be purified and segregated for nanopore research.

## Methods

### Electrophysiology and single channel recording

The electrophysiology setup and nanopore experimental methods have been well-documented^43^. Briefly, the recording apparatus was composed of two chambers (*cis* and *trans*) that were partitioned with a Teflon film. The planar lipid bilayer of 1,2-diphytanoyl-sn-glycerophosphatidylcholine (Avanti Polar Lipids) was formed spanning a 100–150 μm hole in the center of the partition. The α-hemolysin (αHL) protein monomers (Sigma, St. Louis, MO) can be self-assembled in the bilayer to form molecular pores, which can last for hours during electrical recordings. Both *cis* and *trans* chambers were filled with symmetrical 1 M salt solutions (KNO_3_) buffered with 10 mM 3-(N-morpholino)propanesulfonic acid (Mops)^2^ and titrated to pH 7.02. All solutions were filtered before use. DNA oligonucleotides (Table 1) were synthesized and electrophoresis purified by Integrated DNA Technologies (IDT), IA. Before testing, the mixtures of DNA and probes were heated to 90°C for 5 minutes, and then slowly cooled to room temperature. Single-channel currents were recorded with an Axopatch 200A patch-clamp amplifier (Molecular Device Inc., former Axon Inc.), filtered with a built-in 4-pole low-pass Bessel Filter at 5 kHz, and acquired with Clampex 9.0 software (Molecular Device Inc.) through a Digidata 1332 A/D converter (Molecular Device Inc.) at a sampling rate of 20 kHz**·**s^−1^. DNAs were presented in the solution on cis side of the pore (grounded) and a holding potential was applied from the trans side to produce an ion current across the pore. Data was based on at least four separate experiments and obtained by single channel search. The histograms were fitted by exponential log probability (dwell time histogram distribution) or Gaussian function (residual current histogram distribution). The red circles in each figure represent the capturing of DNA duplex in the nanopore. The electrophysiology experiments were conducted at 22 ± 1°C. Data was presented as AVE ± SD (average ± standard deviation).

The ratio of Ag^+^ to DNA duplex was set to 100:1 in all the experiments. Varying the concentration of Ag^+^ (50X, 500X) does not change the number of DNA duplex capturing events significantly. This was similar to the previous findings that the melting temperature reached a plateau when the Ag^+^ concentration was 1.5 fold higher than the DNA^2^. By isothermal titration calorimetry (ITC) and electrospray ionization mass spectrometry measurement, the binding of Ag^+^ to a DNA duplex containing a single C-C mismatch was identified at a 1:1 molar ratio^4^,^10^. The lines under each current trace mark the 0 current.

### Melting temperature measurement

The melting temperatures of duplexes containing C-C, mC-C, or hmC-C mismatches were determined by monitoring the increase in absorbance at 260 nm as a function of temperature (Cary 100 Bio UV-Visible spectrophotometer). The temperature was increased from 4°C to 50°C (for samples without Ag^+^ ion), or from 10°C to 60°C (for samples with Ag^+^), at a rate of 0.5°C/min. P/T_C_ (2/2 μM) and 2 μM Ag^+^ ions were used in the experiment, because previous studies found that the melting temperature reached a plateau when the silver(I) ion concentration was 1.5 fold higher than the DNA^2^. The melting temperature was calculated from the collected data using the Cary WinUV Thermal software. Each sample was repeated at least three times.

### Molecular dynamics simulation

The software NAMD^44^ was used to perform all-atom MD simulation on the IBM bluegene supercomputer. Force fields used in simulations were the CHARMM27^45^ for DNA, the TIP3P^46^ model for water molecules, and the standard one^47^ for ions. Long-range coulomb interactions were computed using the particle-mesh Ewald (PME) method. A smooth (10–12 Å) cutoff was used to compute the van der Waals interaction. After each simulation system was equilibrated at 1 bar, following simulations were carried out in the NVT (*T* = 300 K) ensemble. The temperature of a simulated system was kept constant by applying the Langevin dynamics on Oxygen atoms of water molecules.

## Author Contributions

Y.W. conceived the principal idea and designed the experiments. Y.W. performed the nanopore experiments, collected and analyzed the nanopore data. B.-Q.L. designed and performed the molecular dynamics simulation, collected and analyzed the data. Z.-Y.Y. and K.G. designed and performed the melting temperature measurements, collected and analyzed the data. Y.W., B.-Q.L. and L.-Q.G. wrote the manuscript. All the authors, Y.W., B.-Q.L., Z.-Y.Y., K.G., X.-Y.Z., B.R. and L.-Q.G. discussed the results and commented on the manuscript, and co-wrote the manuscript.

## Supplementary Material

Supplementary Information Support Information

## Figures and Tables

**Table 1:** Sequences of DNAs used in this study

ssDNA	Sequence (5′-3′)
T_C_ (target)	AATAAAATA/**C**/TATAAA
T_mC_ (target)	AATAAAATA/**mC**/TATAAA
T_hmC_ (target)	AATAAAATA/**hmC**/TATAAA
P (probe)	TTTATA**C**TATTTTATT
P′ (probe)	TTTATA**C**TATTTTATTAGAAAAAAAAAAGAAAAAAAAAAAAAAA

**Table 2:** Calculation of *K*_d_ with and without Ag^+^ (See detailed description at “Nanopore measurement of double-stranded DNA equilibrium constant” in Supplementary Information)

	ss T_C_ *K*_on_ (uM^−1^s^−1^)^a^	ss P *K*_on_ (uM^−1^s^−1^)^a^	ssDNA frequency in the mixture of T_C_ and P (s^−1^)^b^	ssDNA frequency in the mixture of T_C_ and P with Ag^+^ (s^−1^)^b^	*K*_d_, uM (−Ag^+^)^c^	*K*_d_, uM (+Ag^+^)^c^
	3.60	3.31	6.21	3.81	0.11	0.039
	3.41	3.78	6.25	4.20	0.11	0.046
	3.73	3.33	6.57	4.23	0.12	0.047
	3.88	3.36	7.03	4.17	0.14	0.045
	3.80	3.02				
		3.84				
AVE ± SD	3.68 ± 0.19	3.49 ± 0.31	6.52 ± 0.38	4.10 ± 0.19	0.12 ± 0.01	0.04 ± 0.004

*^a^*: *k_on_*, capture rates for ssDNAs T_C_ and P.

*^b^*: *f_ss_*, frequency for ssDNA translocation events in the mixture of ssDNA T_C_ and P (8 μM/8 μM).

*^c^*: *K_d_*: equilibrium dissociation constant calculated from *k_on_* for T_C_ and P1 and *f_ss_* using Eq.S3. When *K*_d_ were calculated, the averaged trapping rates (*K*_on_) for ssDNA T_C_ and P were used.
