# Supplementary material for: Single Molecule Investigation of Ag+ Interactions with Single Cytosine-, Methylcytosine- and Hydroxymethylcytosine-Cytosine Mismatches in a Nanopore
Source: Sci Rep. 2014 Aug 8;4:5883. doi: 10.1038/srep05883 (PMC4126007; doi:10.1038/srep05883)
Supplement: Supplementary Information — Support Information [file srep05883-s1.doc]

**Supplementary Information**

**Single Molecule Investigation of Ag^+^ Interactions with Single Cytosine-, Methylcytosine- and Hydroxymethylcytosine-Cytosine Mismatches in a Nanopore**

Yong Wang, Bin-Quan Luan, Zhiyu Yang, Xinyue Zhang, Brandon Ritzo,

Kent Gates, and Li-Qun Gu

**S1. Nanopore measurement of double-stranded DNA equilibrium constant**

We assume that the nanopore has no influence on the equilibrium constant of the reaction in the buffer solution. As shown in the following reaction scheme, the hybridization of single-stranded target (T) and probe (P) forms a double-stranded complex (T•P). The binding of Ag+ to the C-C mismatch in the T•P hybrid (T•P•Ag) can stabilize the dsDNA. The new equilibrium results in a decrease in single-strand (T and P) concentrations, and an increase in dsDNAs (T•P and T•P•Ag) concentrations.

T+P T•PT•P•Ag

The equilibrium constant *K* can be obtained from the change in ssDNA translocation events in the nanopore. The capture rates for T and P in the nanopore are *k_on_*(T) and *k_on_*(P). T and P have the same initial concentration *C*_0_ of 8 µM, and equilibrium concentration *C*. The frequency of total translocation events for un-hybridized T and P is *f_ss_*.

*f_ss_* = *k_on_*(T)·*C*+ *k_on_*(P)·*C* (S1)

Thus

*C* = *f_ss_*/[*k_on_*(T)+ *k_on_*(P)] (S2)

The total dsDNA concentration is C_0_-C. Therefore *K* can be determined as

*K* = *C*^2^ /(*C*_0_-*C*) (S3)

In our study, *k_on_*(T) and *k_on_*(P) were obtained from ssDNA T and P translocation experiments. *k_on_*(T) was 3.68±0.19 µM^-1^·s^-1^ and *k_on_*(P) P was 3.49±0.31 µM^-1^·s^-1^. Measured *f_ss_* was 6.52±0.38 s^-1^. Thus from Eq. S2, *C* was calculated to be 0.91 µM, and total dsDNA concentration was 8-0.91=7.09 uM. Finally from Eq. S3, *K* was calculated to be 0.12±0.01 µM^-1^. In the presence of Ag^+^, *f_ss_* was 4.10 ± 0.19 s^-1^, thus *C* = 0.57 µM, and *K* in the presence of Ag^+^ was increased to 0.04±0.004 µM^-1^. With the addition of silver ions, the *K* decreased about 3 fold, which suggest a tighter binding for this dsDNA∙Ag^+^ compared to dsDNA itself.

**Notes and Movie descriptions**

Note S1: At first, we were trying to use P’ (sequence in Table 1) as the probe, because studies have found that when the probe was attached with an overhang, the capture rate can be greatly increased with a shorter unzipping time[^1^](#_ENREF_1). When P’ was hybridized with T_C_ (sequence in Table 1), the unzipping was very fast since there was a C-C mismatches in the duplex, and the unzipping events were in the range of 0.5 ms – 10 ms. Events of this duration cannot be distinguished from the ssDNA P because ssDNA itself can generate long events from 1ms to 10 ms in KNO_3_. Therefore, we set the 10 ms as the cutoff point for DNA duplex capturing. When we used P to hybridize with T_C_, the observed dwell time increased, allowing us to distinguish the signal from that of ssDNA.

Note S2: In the nanopore recording, we considered events longer than 10ms as the DNA duplex capturing. We indentify 50%-60% DNA duplexes trapping events (>10ms) with an ending spike (Figure 2a1,b1), which was reported as unzipping signature in the nanopore[^1^](#_ENREF_1). We can see that there are some downward flicks (Figure 2a1,b1) when the dsDNA with blunt ends (no overhang) was trapped in the nanopore. The detailed molecule configurations were not discussed here. Similar phenomenon has been reported that DNA hairpins with a duplex blunt ending generate two main conductance states[^2-4^](#_ENREF_2).

Note S3: We see two residual current peaks for mC-C with Ag^+^ (33.9 pA and 38.1 pA, Figure 3d), but only a single peak for C-Ag-C (Figure 2d, red). This may caused by the weak interaction between Ag^+^ and mC-C mismatches, or a portion of the mC-C duplexes that did not bind to the Ag^+^, which correspond to the 38.1pA residual current. Molecular dynamics (MD) simulations indicate that hydrogen bonds are alternatively formed between N4_A_ and N3_B_ atoms and between N3_A_ and N4_B_ atoms, and there is a 2.6 fold difference in binding energy between these two conformations of Ag^+^ binding to C-C mismatch. Note that mC-C mismatches has a similar residual current at 37.4 pA (Figure 3d), which is very close to 38.1 pA.

Note S4: We adopt the force field for Ag^+^ that was characterized for Ag^+^ in water[^5^](#_ENREF_5). The force field for the interaction between Ag^+^ and a biomolecule is still not well developed. In our MD simulation of Ag^+^ in a duplex with a C-C mismatch, we adopted the force field: ε_Ag+/N3_ = 0.218 kcal/mol; ε_Ag+/O2_ = 0.169 kcal/mol; σ_Ag+/N3_ = 0.227 nm; σ_Ag+/O2_ = 0.227 nm. As shown in Fig. S8, the mean distance between Ag^+^ and a N3 atom in a binding state is about 0.206 nm, consistent with the distance found in the crystal structure (PDB: 2KE8).

Movie **S1:** A trajectory of the MD simulation of a DNA duplex with a C-C mismatch.

Movie **S2:** A trajectory (same as shown in movie S1) of the MD simulation of a DNA duplex with a C-C mismatch, coordinated with a K^+^.

Movie **S3:** A trajectory of the MD simulation of a DNA duplex with a C-C mismatch, stabilized with a bound Ag^+^.

Movie **S4:** A trajectory of the MD simulation of a DNA duplex with a mC-C mismatch.

Movie **S5:** A trajectory of the MD simulation of a DNA duplex with a hmC-C mismatch.

**REFERENCES**

1. Wang, Y.; Zheng, D.; Tan, Q.; Wang, M. X.; Gu, L. Q. Nanopore-Based Detection of Circulating Micrornas in Lung Cancer Patients. *Nature nanotechnology* 2011, 6, 668-74.

2. Vercoutere, W. A.; Winters-Hilt, S.; DeGuzman, V. S.; Deamer, D.; Ridino, S. E.; Rodgers, J. T.; Olsen, H. E.; Marziali, A.; Akeson, M. Discrimination among Individual Watson-Crick Base Pairs at the Termini of Single DNA Hairpin Molecules. *Nucleic acids research* 2003, 31, 1311-8.

3. Vercoutere, W.; Winters-Hilt, S.; Olsen, H.; Deamer, D.; Haussler, D.; Akeson, M. Rapid Discrimination among Individual DNA Hairpin Molecules at Single-Nucleotide Resolution Using an Ion Channel. *Nature biotechnology* 2001, 19, 248-52.

4. DeGuzman, V. S.; Lee, C. C.; Deamer, D. W.; Vercoutere, W. A. Sequence-Dependent Gating of an Ion Channel by DNA Hairpin Molecules. *Nucleic acids research* 2006, 34, 6425-37.

5. Spezia, R.; Nicolas, C.; Archirel, P.; Boutin, A. Molecular Dynamics Simulations of the Ag+ or Na+ Cation with an Excess Electron in Bulk Water. *J Chem Phys* 2004, 120, 5261-8.

**Figure S1**

**b**

open current

0 pA

residual current

dwell time


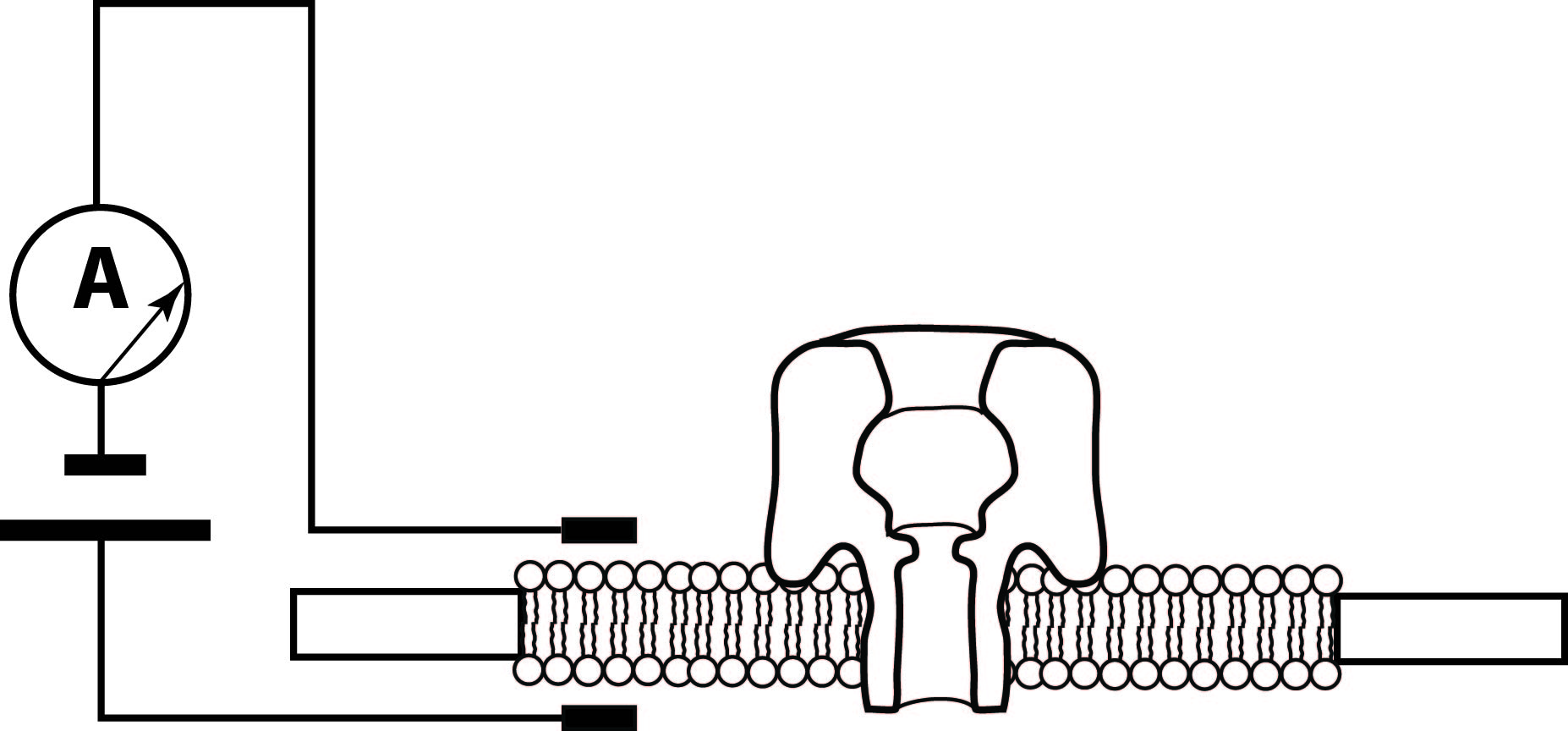


NO_3_^-^

K^+^

KNO_3_

KNO_3_

cis

trans


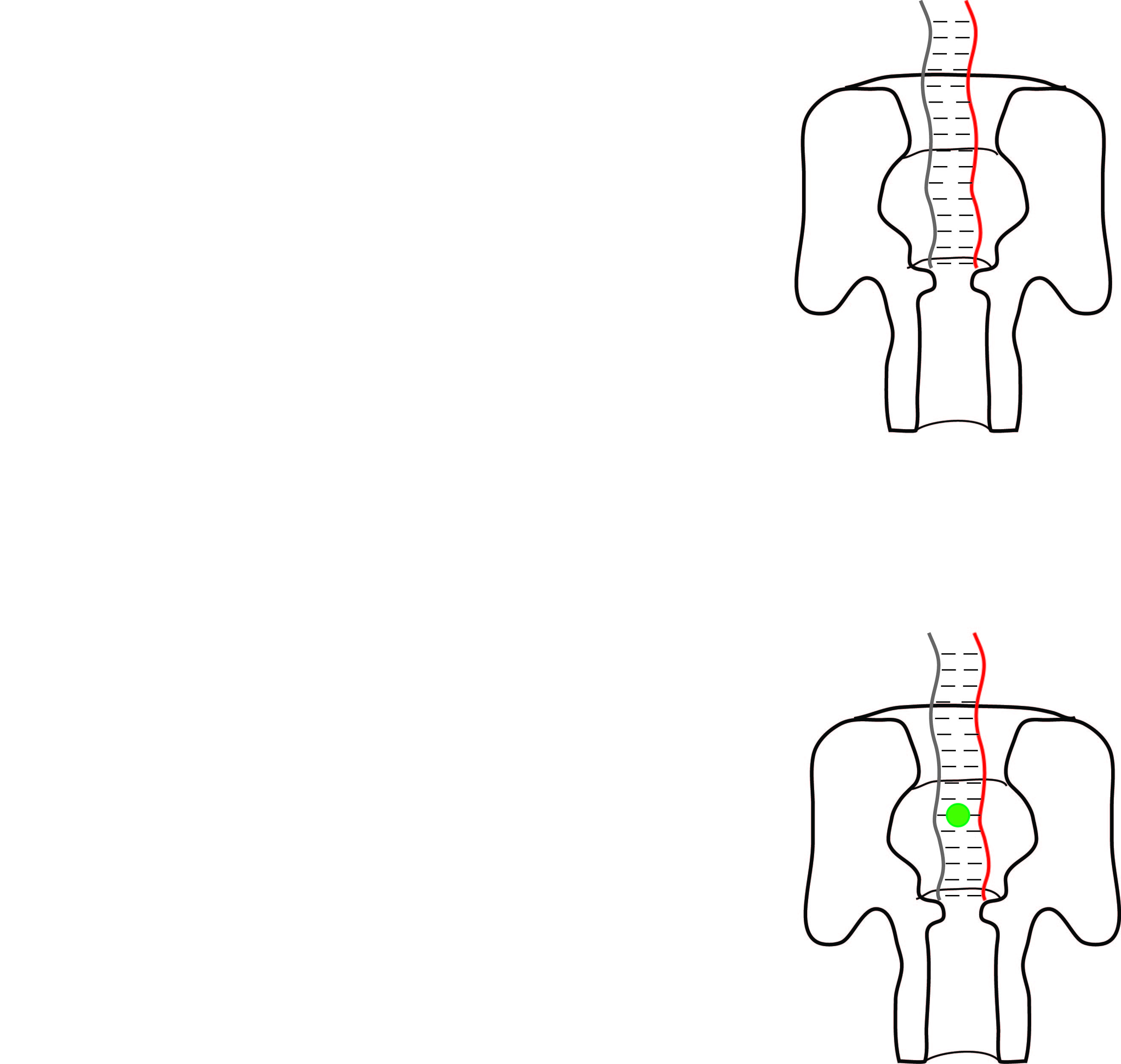


Nanocavity

1.4 nm constriction site

2.6 nm opening

**a**

t_off_

t_on_

**Figure S1: The nanopore recording platform. a**, the alpha-hemolysin nanopore has a nanocavity (2.6nm opening and a 1.4nm constriction site) can capture and hold the DNA duplex, **b**, during nanopore recording, a single α-HL nanopore is inserted into a lipid bilayer that separates two chambers (termed cis and trans) containing KNO_3_ buffer solution. Ionic current through the nanopore was carried by K^+^ and NO_3_^-^, ions, and a patch clamp amplifier applies voltage and measures ionic current. **c**, when a molecule interacts with the nanopore which will block the ionic pathway, then generate a “block” event. From the dwell time and residual current we can obtain meaningful information of the interactions between the molecule and the nanopore.

**Figure S2**


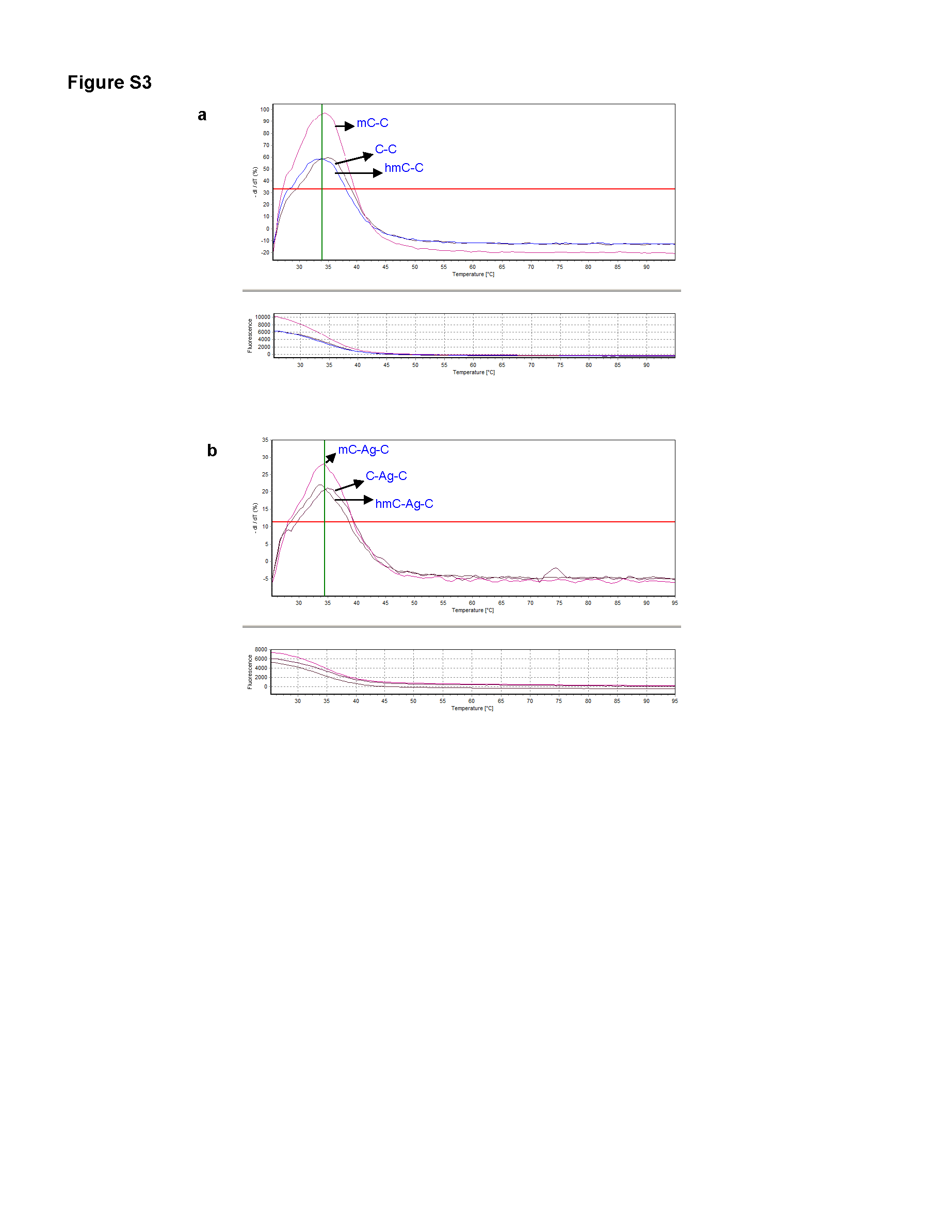

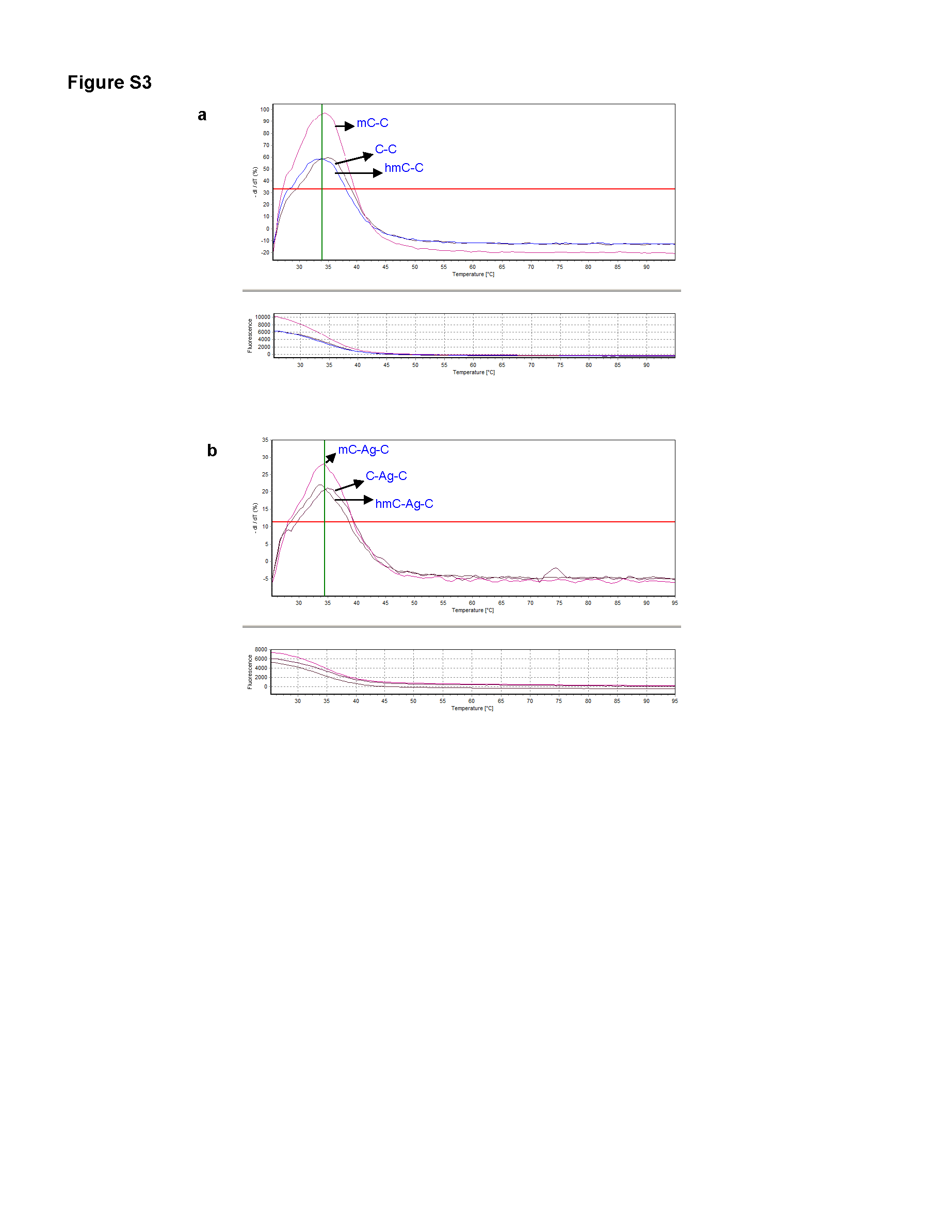


**Figure S2: The Histograms for the t_on_ distribution for C-C duplex.** a, without Ag^+^ and b, with the addition of Ag^+^. The definition of t_on_ was shown in Supplementary Figure S1.

**Figure S3**

hmC-C

mC-C

C-C


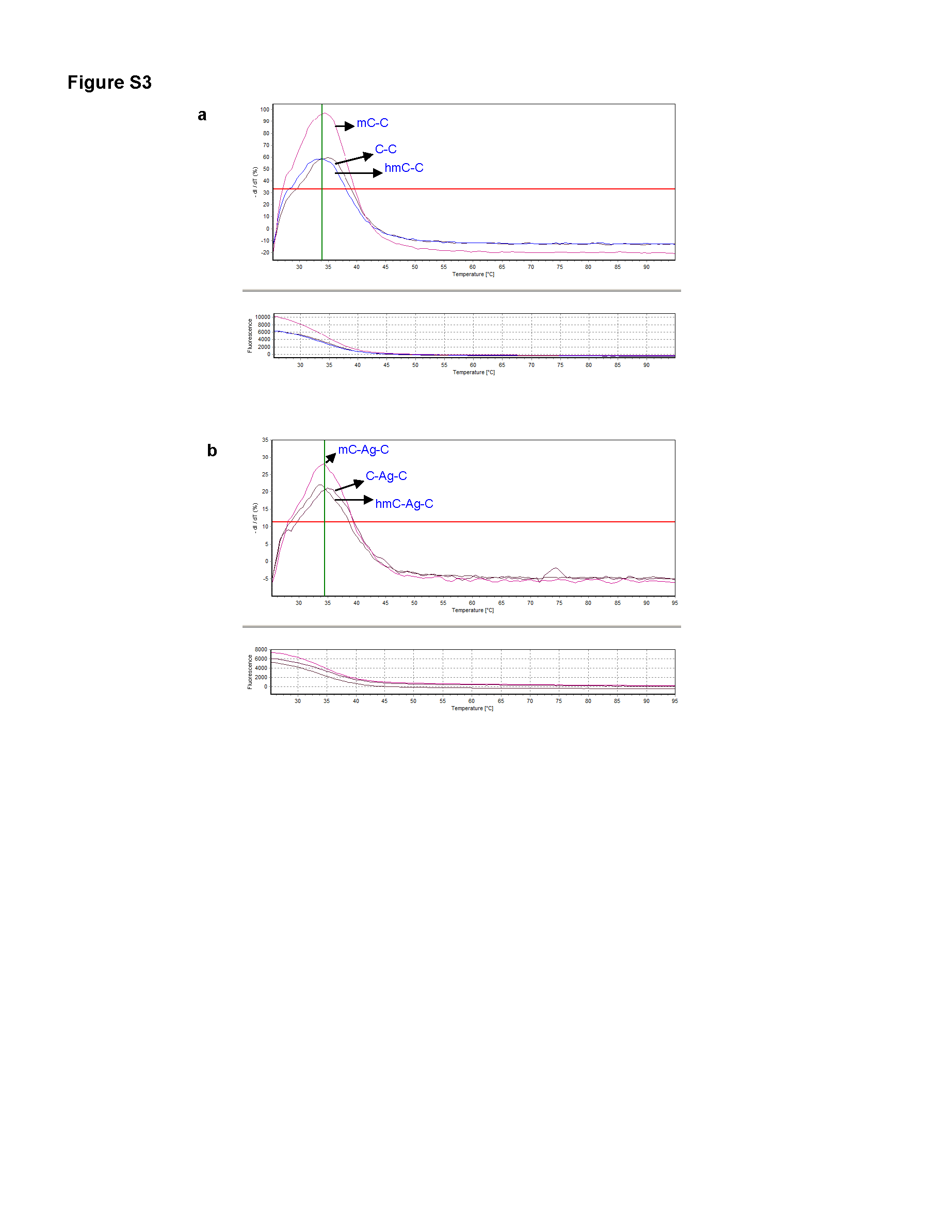


**
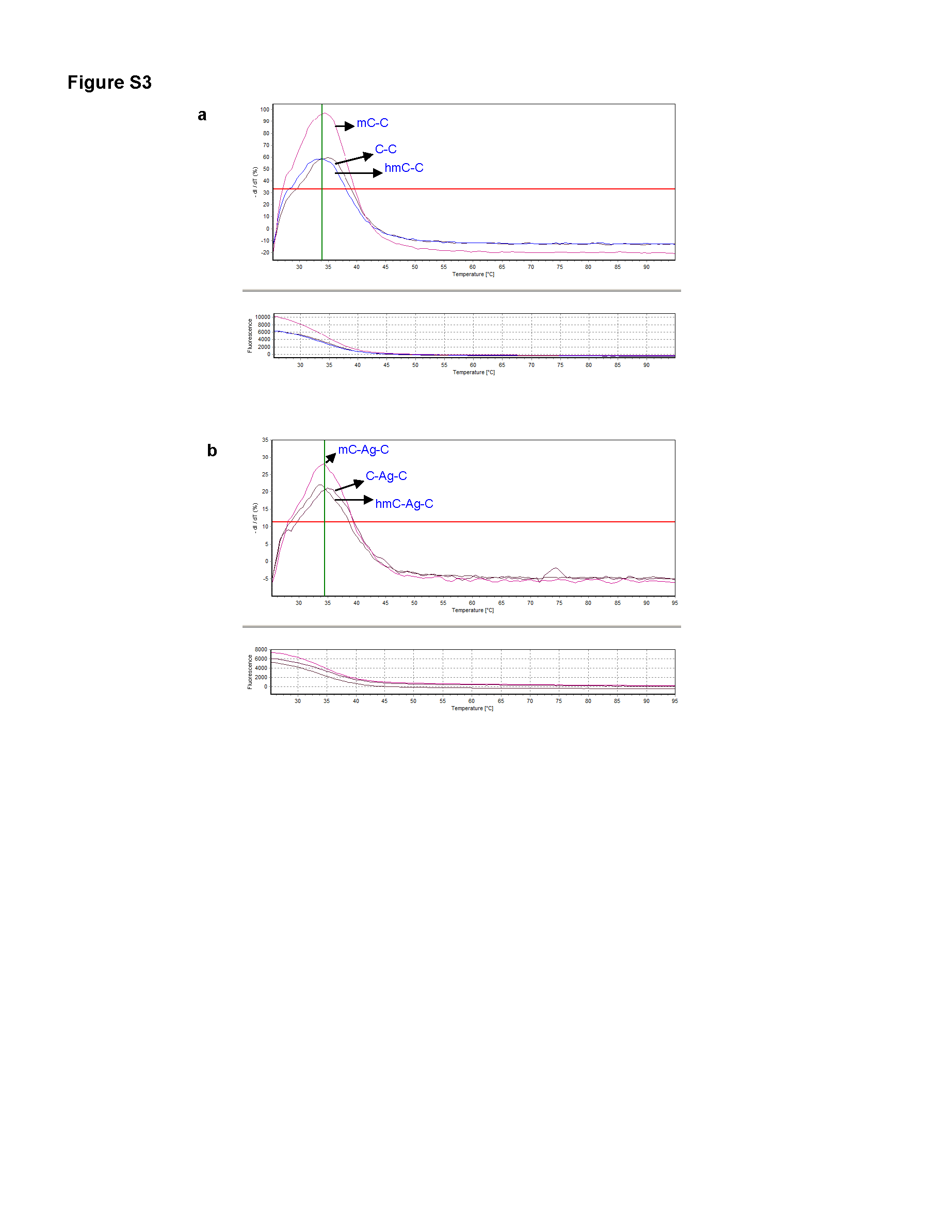
**

**Figure S3: The addition of Ag^+^ decreased the residual current at different degrees for C-C and mC-C mismatches, but has no effect on hmC-C**. **a**, C-C generated a peak of 41.5 ± 0.4 pA, C-Ag-C generated a peak of 36.8±0.2 pA. Similarly, the residual currents for mC-C was 37.4±0.7 pA; for mC-Ag-C was 33.9±0.8 pA; for hmC-C was 36.3±0.95 pA; for hmC-Ag-C was 36.2±0.71 pA. **b**, The differences of residual current for duplexes C-C(mC, hmC) *minus* C-Ag-C(mC, hmC). They are 4.7±0.4 pA, 3.5±1.1 pA and 0.1±1.19 pA respectively. The residual current differences with the addition of Ag^+^ is C-C > mC-C > hmC-C. See main text for details.

**Figure S4**


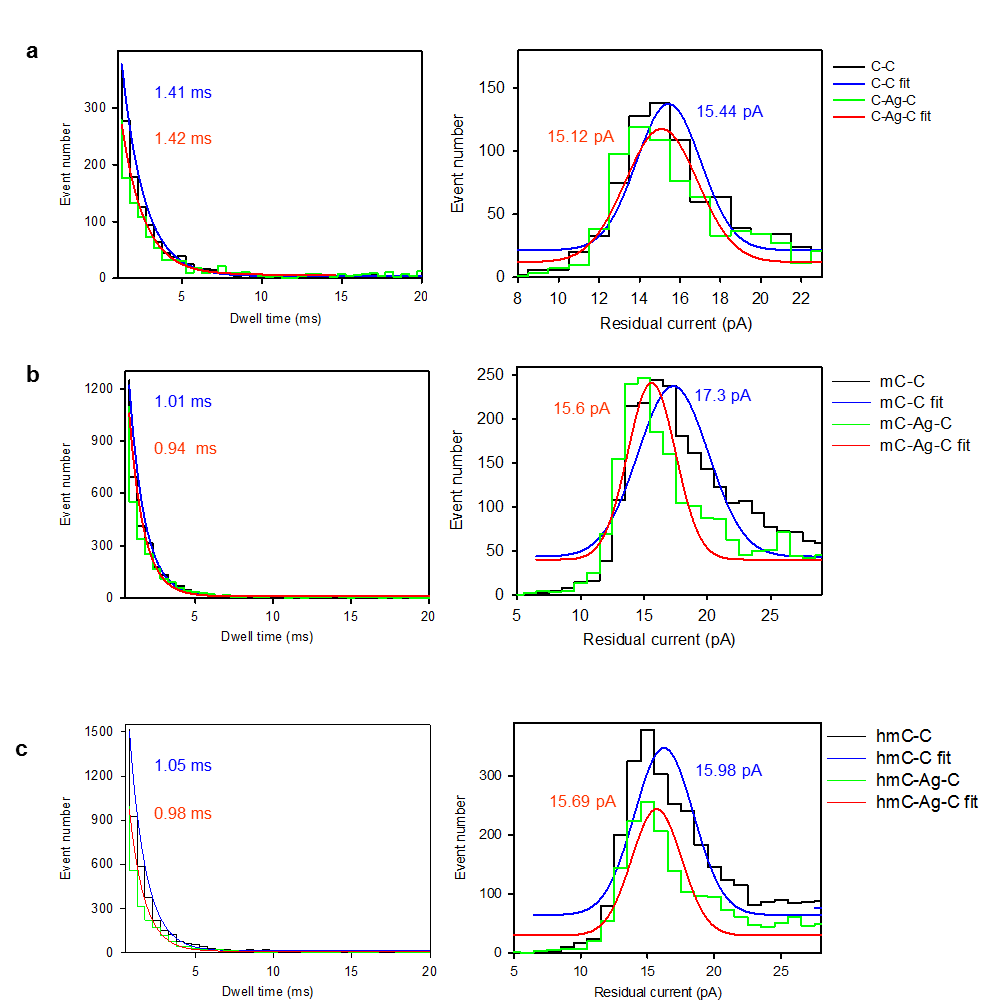


**Figure S4: Ag^+^ doesn’t interact with ssDNAs T_C_, T_mC_ or T_hmC_. a,** The un-hybridized ssDNAs (when ssDNA T_C_ hybridized with P) with (blue) and without (red) Ag^+^ in the nanopore. Left panel: the histogram of the dwell time. Right panel: the histogram of residual. **b**, The un-hybridized ssDNAs (when ssDNA T_mC_ hybridized with P) with (blue) and without (red) Ag^+^ in the nanopore. Left panel: the histogram of the dwell time. Right panel: the histogram of residual currents. **c**, The un-hybridized ssDNAs (when ssDNA T_hmC_ hybridized with P) with (blue) and without (red) Ag^+^ in the nanopore. Left panel: the histogram of the dwell time. Right panel: the histogram of residual currents. In **a**, **b** and **c** we can indentify similar dwell times and residual currents. These values were very similar to that generated by ssDNA P (see **Figure 1**).

**Figure S5**


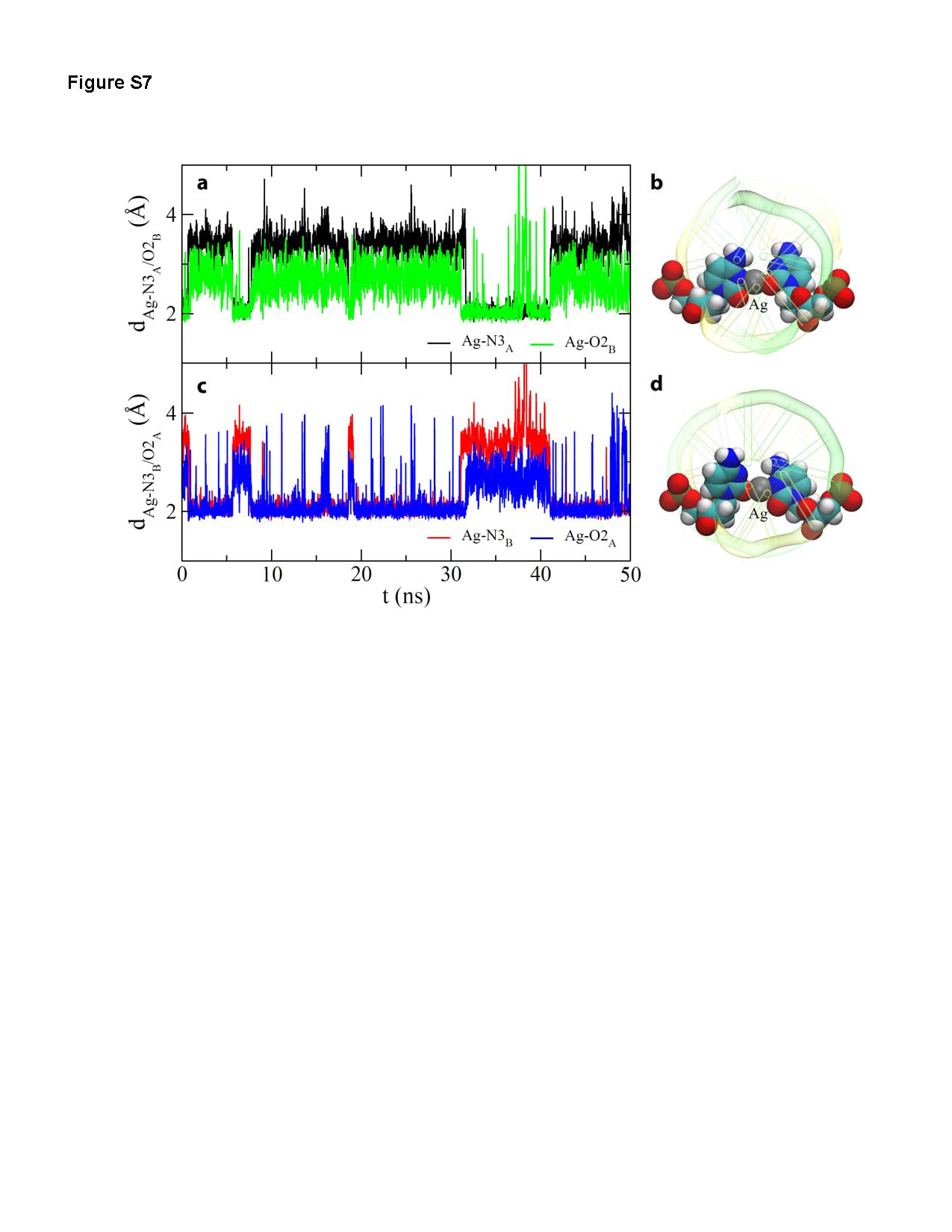


**Figure S5: MD simulation of a DNA duplex with the C-C mismatch that is coordinated with a Ag^+^.** **a**, Distances between the Ag^+^ and N3_A_ (black) or between Ag^+^ and O2_B_ (green). In a binding state, these distances are about 2.06 Å. **b**, A snap-shot of a corresponding binding state from the simulation. **c**, Distances between the Ag^+^ and N3_B_ (red) or between Ag^+^ and O2_A_ (blue). In a binding state, these distances are about 2.06 Å. **d**, A snap-shot of a corresponding binding state from the simulation. These results show that for a Ag^+^ there are two symmetric binding states (**b** and **d**) that are alternatively present in the simulated structure (**a** and **c**).

**Figure S6**


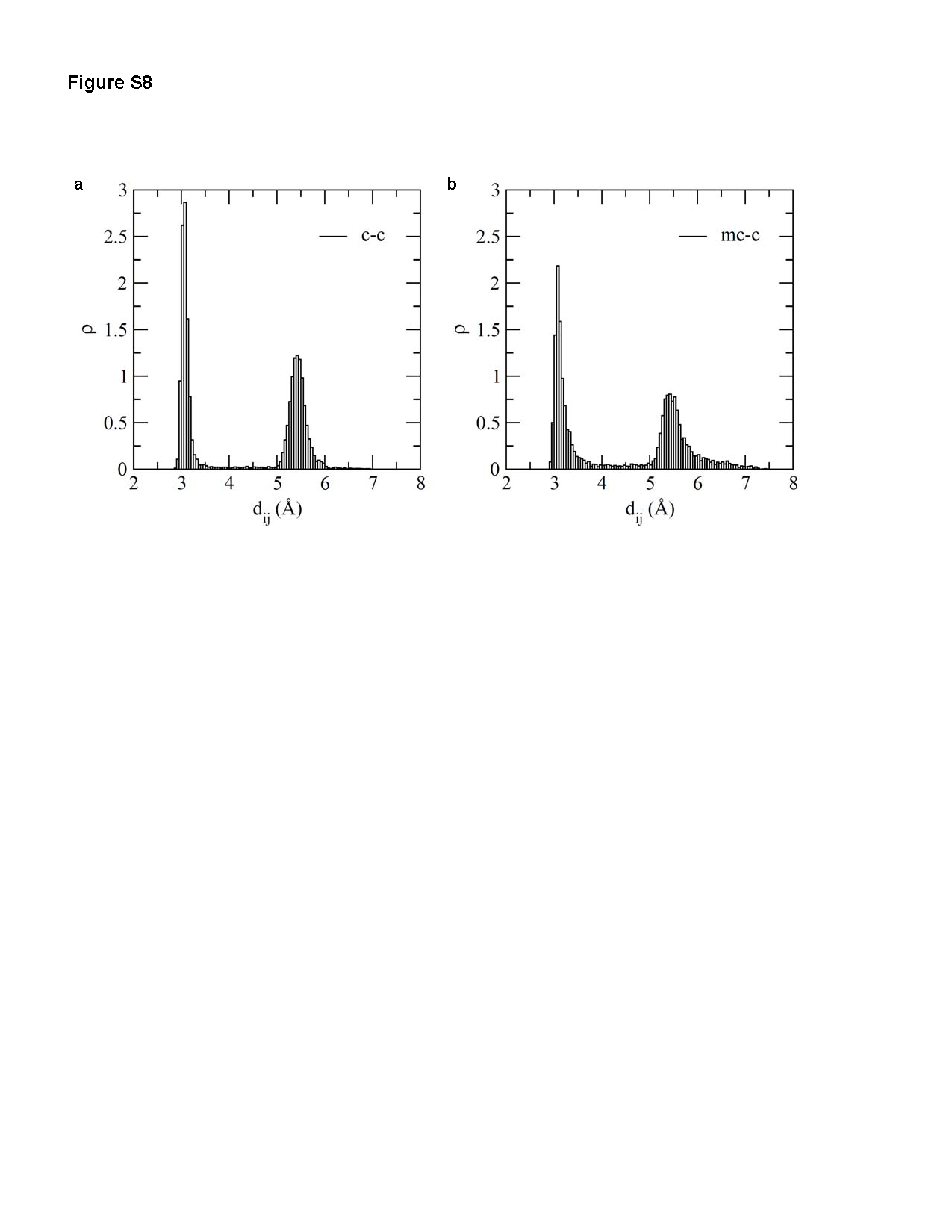


**Figure S6: Probability densities of hydrogen-bond lengths between N3 and O2 atoms of difference bases in a mismatched pair. a**, the mismatched pair is C-C. **b**, the mismatched pair is mC-C. The sharper peak in **a** indicates that the hydrogen-bond mediated base-pairing is more stable in the C-C mismatch.

**Figure S7**


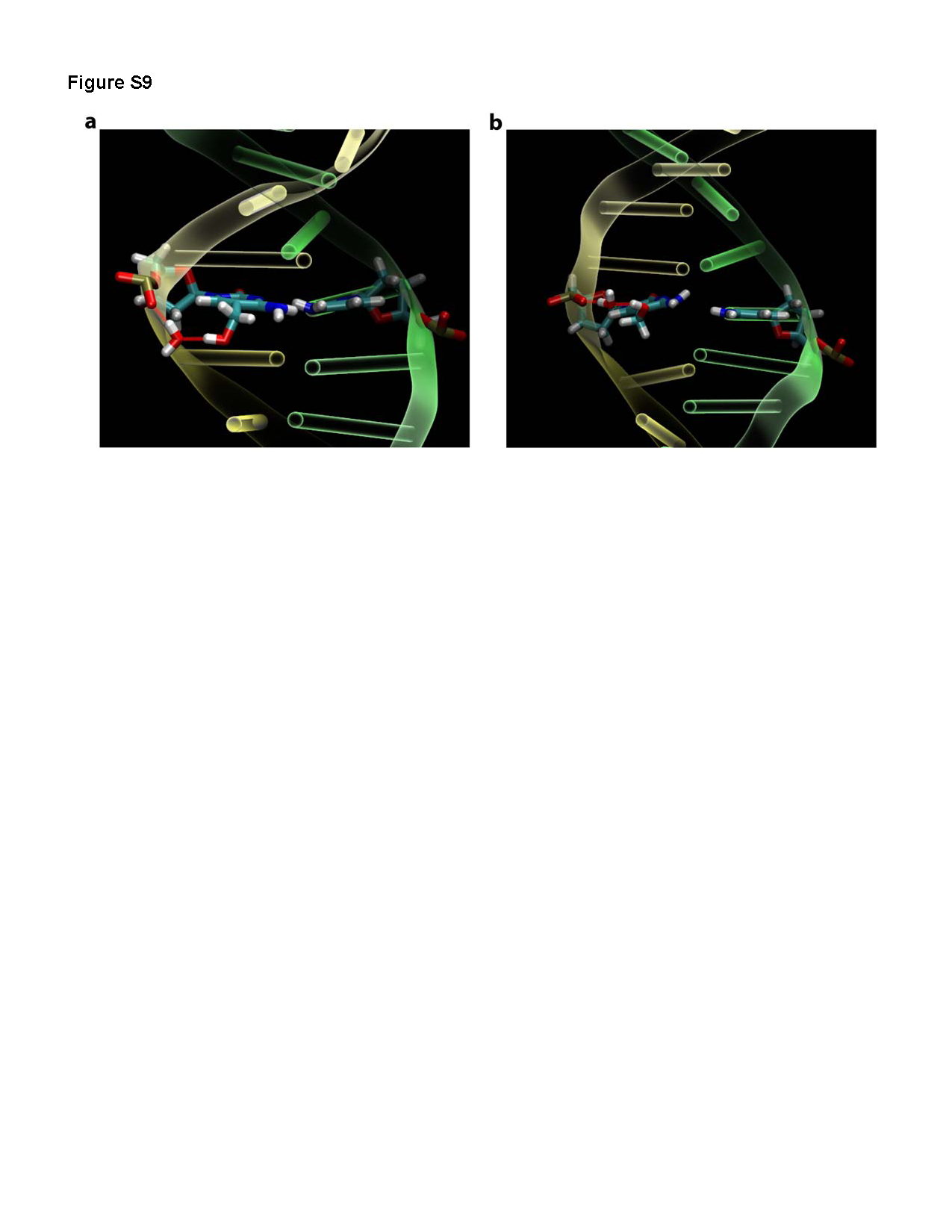


**Figure S7: The role of the hydroxyl group in the hmC. a** and **b,** Two examples of water mediated interaction between the phosphate group and the hydroxyl group in the hmC. The water molecule forms hydrogen bonds with both the phosphate group in the DNA backbone and the hydroxyl group in the hmC. Additionally, as shown in Fig. 3**c**, it is possible to form a direct interaction, via. the hydrogen bond, between the phosphate group and the hydroxyl group.

171 ms

59 ms

**Figure S8**

**Figure S8: The dwell time distribution of dsDNA duplex C-C (ssDNA T_C_ hybridized with P) with the addition of Ag^+^ can not be fitted by 1 component.** We fitted the histogram by exponential log probability, 2 components, which fitted the histogram very well (Figure 2C, red).
